# An eye hospital’s humanitarian response to COVID-19

**Published:** 2020-09-01

NIRPHAD (Naujhil Integrated Rural Project for Health and Development) Rural Eye Hospital is a secondary eye hospital in the state of Uttar Pradesh, Northern India. It focuses mainly on eye patients from vulnerable populations and has strong links to community-based rehabilitation services, as well as organisations of people with disabilities.

**Figure 1:**
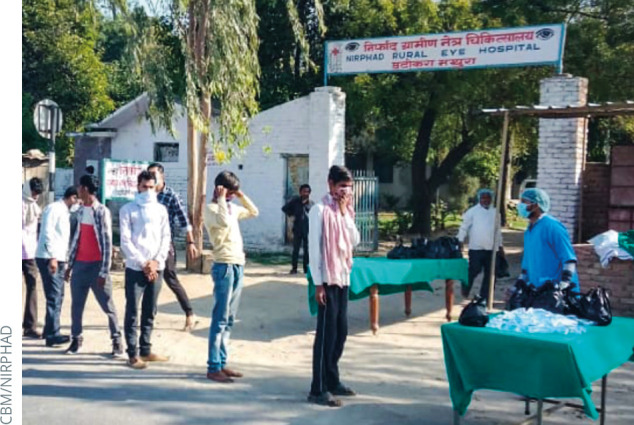
NIRPHAD Rural Eye Hospital distributed sanitation packs to people queueing in front of the hospital’s entrance

Since the declaration of the COVID-19 pandemic, the eye hospital offered services to emergency patients only. At the same time, staff members decided to organise humanitarian response activities. NIRPHAD Rural Eye Hospital is located next to one of the main national highways in India, and the sudden announcement of a national lockdown in India at the end of March resulted in thousands of migrant workers passing by on their way back to their homes in rural villages. Hospital staff members handed out around 1,500 sanitisation kits and food packs in this time (Figure 1).

**Figure 2:**
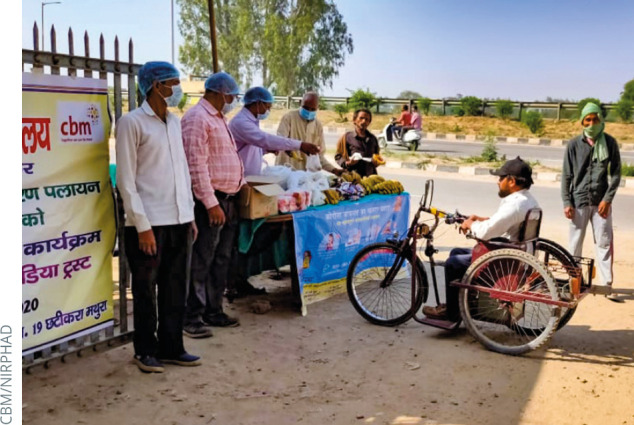
NIRPHAD Rural Eye Hospital distributed food and sanitation packs to a man with a physical disability who is using a three-wheeled vehicle

**Figure 3:**
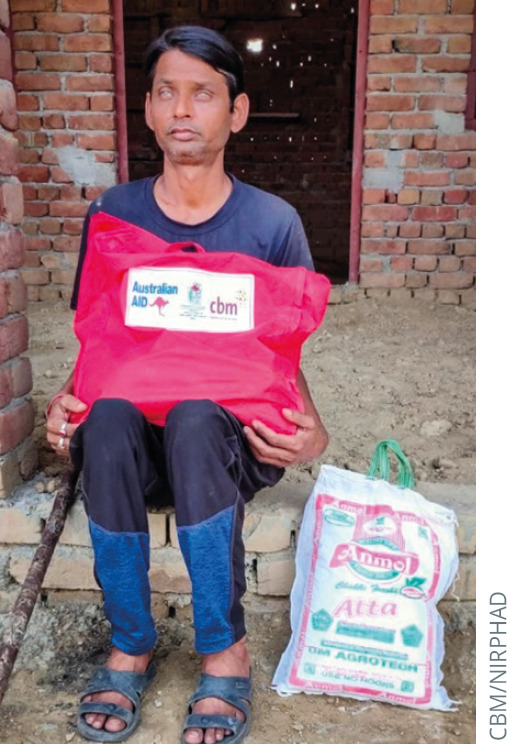
A man with visual and physical impairment received food and a sanitation kit

Eye hospital personnel also started to distribute soap, masks and food to poor and vulnerable people in Mathura town, focusing on people with disabilities (Figures 2 and 3). This targeted humanitarian response was possible because the health workers already had access to information about people with disabilities in the community, including where they live, thanks to a disability-disaggregated community survey that was conducted before the pandemic.

Personnel trained in disability-inclusive development supported the district authorities to provide accessible health information, for example by using plain language that is understood easily by everybody, including people with cognitive disabilities.

*We would like to acknowledge Mr Shashikant Mishra and Mr Jeetesh Lavanya from the Naujhil Integrated Rural Project for Health and Development, as well as Mr Shakeeb Khan and Mr Nirad Bag from the CBM India Trust*

